# Review: seizure-related consolidation and the network theory of epilepsy

**DOI:** 10.3389/fnetp.2024.1430934

**Published:** 2024-08-22

**Authors:** Mark R. Bower

**Affiliations:** Department of Neurology, Yale University, New Haven, CT, United States

**Keywords:** sleep, memory consolidation, neural assemblies, epilepsy, systems consolidation, epileptogenesis

## Abstract

Epilepsy is a complex, multifaceted disease that affects patients in several ways in addition to seizures, including psychological, social, and quality of life issues, but epilepsy is also known to interact with sleep. Seizures often occur at the boundary between sleep and wake, patients with epilepsy often experience disrupted sleep, and the rate of inter-ictal epileptiform discharges increases during non-REM sleep. The Network Theory of Epilepsy did not address a role for sleep, but recent emphasis on the interaction between epilepsy and sleep suggests that post-seizure sleep may also be involved in the process by which seizures arise and become more severe with time (“epileptogenesis”) by co-opting processes related to the formation of long-term memories. While it is generally acknowledged that recurrent seizures arise from the aberrant function of neural circuits, it is possible that the progression of epilepsy is aided by normal, physiological function of neural circuits during sleep that are driven by pathological signals. Studies recording multiple, single neurons prior to spontaneous seizures have shown that neural assemblies activated prior to the start of seizures were reactivated during post-seizure sleep, similar to the reactivation of behavioral neural assemblies, which is thought to be involved in the formation of long-term memories, a process known as Memory Consolidation. The reactivation of seizure-related neural assemblies during sleep was thus described as being a component of Seizure-Related Consolidation (SRC). These results further suggest that SRC may viewed as a network-related aspect of epilepsy, even in those seizures that have anatomically restricted neuroanatomical origins. As suggested by the Network Theory of Epilepsy as a means of interfering with ictogenesis, therapies that interfered with SRC may provide some anti-epileptogenic therapeutic benefit, even if the interference targeted structures that were not involved originally in the seizure. Here, we show how the Network Theory of Epilepsy can be expanded to include neural plasticity mechanisms associated with learning by providing an overview of Memory Consolidation, the mechanisms thought to underlie MC, their relation to Seizure-Related Consolidation, and suggesting novel, anti-epileptogenic therapies targeting interference with network activation in epilepsy following seizures during post-seizure sleep.

## Introduction

The Network Theory of Epilepsy (NTE) proposed that seizure initiation arises from the interactions of multiple brain structures, rather than originating in restricted areas of damaged tissue (i.e., a seizure “focus”) (Spencer, 2002)⁠. While there are reasonable arguments that seizure initiation (“ictogenesis”) may be focal (Schevon, CA in Zaveri et al., 2020)⁠ and surgical resection of focal, damaged brain tissue (“lesions”) is often an effective therapy against epilepsy (Engel, 2018)⁠, multiple lines of evidence suggest epilepsy is a network disease and that ictogenesis utilizes existing brain networks (Beenhakker and Huguenard, 2009; Zaveri, H in Zaveri et al., 2020)⁠, which is supported both theoretically (Ponten et al., 2007; Reijneveld et al., 2007) and anatomically (Lopes da Silva et al., 2003; Schindler et al., 2007)⁠. When NTE was proposed, theories of ictogenesis favored a role for static, focal, “sick” tissue whose properties predisposed that tissue to produce pathological, rhythmic activity. The “Epileptic Neuron” theory proposed that a fixed group of damaged neurons possessed specific cellular features that promoted uncontrolled, rhythmic activity, making them the initiation site for seizures (Ward et al., 1956)⁠. *In vivo* single neuron recordings in patients undergoing intracranial recording for the treatment of epilepsy, however, failed to identify neurons whose activity reliably changed prior to the start or at the initiation of seizures (Verzeano et al., 1971; Babb and Crandall, 1976; Wyler et al., 1982; Babb et al., 1987)⁠. In regards to damaged circuits, the “Dentate as a Gate” theory proposed that damage to the dentate gyrus of hippocampus allowed epileptiform activity to pass downstream to hippocampal subfields (e.g., CA1, CA3), thus initiating temporal lobe seizures (Lothman et al., 1992)⁠. The “Mossy Cell Loss-Induced Sprouting” (Lynch and Sutula, 2000; Nadler, 2003; Scharfman et al., 2003)⁠, “Dormant Basket Cell” (Sloviter, 1991)⁠ and “Irritable Mossy Cell” (Santhakumar et al., 2000)⁠ hypotheses each described different mechanisms producing persistent changes in local circuit inhibition or excitation that could lead to reduced thresholds for dentate granule cells leading to seizure initiation. Simultaneous, *in vivo* recording of multiple neurons (ensemble recordings) in dentate gyrus of pilocarpine-treated rats, however, revealed heterogeneous changes in firing rates prior to seizures, where the firing rate of a given neuron could increase, decrease, or remain unchanged and that this response could change with subsequent seizures (Bower and Buckmaster, 2008)⁠. These variable responses challenged static, focal theories of ictogenesis, but were consistent with a theory of network-driven ictogenesis, like NTE.

NTE did not address directly the mechanisms by which epilepsy originates and becomes more severe (epileptogenesis) and there are fewer theories of epileptogenesis than ictogenesis (Bragin et al., 2000; Hsu et al., 2008; Pitkänen et al., 2015; Koepp et al., 2024)⁠. The question of whether seizures, themselves, promote epileptogenesis (“Do seizures beget seizures?”) remains unresolved (Jiruska et al., 2023)⁠. While some physiological changes associated with the emergence of epilepsy are known to occur in specific brain structures (e.g., Yamada and Bilkey, 1991; Yaari and Beck, 2002)⁠, epilepsy is also associated with physiological changes in multiple brain structures (Sutula et al., 2003; Saniya et al., 2017; Chauhan et al., 2022), although the cause and effect relationship between anatomical changes and epileptogenesis remains unclear⁠. It is noteworthy that such changes are normally associated with increasing severity of epilepsy, as remission of epilepsy is linked to control of seizures by medication and/or surgery (Sperling, 2004)⁠. It is also noteworthy that these persistent, widespread, physiological changes constitute a form of neural plasticity that is associated with the progression of epilepsy and that such neural plasticity mechanisms are thought to be related to active processes that occur during sleep (Goddard and Douglas, 1975; Beenhakker and Huguenard, 2009; Halász and Szűcs, 2020).

Neural plasticity has been defined as “lasting structural and functional changes in neurons in response to a stimulus (such as an experience)” (Walker and Stickgold, 2004)⁠. One form of neural plasticity that has been studied for decades and possesses a rich literature is Memory Consolidation (MC), a process involved in the formation of long-term memories through processes that occur during sleep (McGaugh, 2000)⁠. MC is a network phenomenon involving the synchronization of multiple brain structures, including hippocampus, thalamus and neocortex, and recent work has detailed how MC can be observed at the cellular level through the discovery of “engram neurons” (Saniya et al., 2017; Guskjolen and Cembrowski, 2023)⁠, which are defined as “neurons that are preferentially involved in the encoding, consolidation, and retrieval of a particular memory” (Josselyn and Tonegawa, 2020)⁠. Such engrams, however, would not be expected to produce network-wide changes that would be observable in gross anatomical changes that could be observed directly through imaging. Rather, it is reasonable to assume that the quantitative measurement of functional changes in neural circuits would require the use of statistical techniques sensitive to such changes, which will be discussed later in this review. Because MC involves multiple brain structures, linking epilepsy with sleep-related neural plasticity mechanisms would extend NTE to include the network interactions that occur across multiple brain structures during sleep, thus expanding NTE to include other aspects of epilepsy including epileptogenesis. As with engrams associated with behavioral learning and though detectable brain lesions are associated with better surgical resection outcomes, many patients with epilepsy display no imaging lesions or abnormalities (Téllez-Zenteno et al., 2010), so it is reasonable to apply the same statistical techniques used in behavioral learning to epilepsy-related neural plasticity⁠. As can be seen from the multiple fields of study that have advanced our understanding of the network basis of MC, understanding how MC could relate to epilepsy, in general, and epileptogenesis, in particular, would benefit from viewing consolidation from multiple perspectives: behavioral, sleep, systems, cellular, anatomical, neural plasticity and theoretical, to name a few. In this review, we can only touch on these many perspectives in an overview of aspects of MC related to epilepsy, describe some neural plasticity mechanisms that are thought to underlie MC, and present evidence that these mechanisms might be co-opted by pathological, seizure-related activity to produce Seizure-Related Consolidation (SRC), raising the possibility of extending NTE to encompass both ictogenesis and epileptogenesis.

## Memory consolidation: behavior, theory and anatomy

Memory Consolidation (MC) describes the behavioral observations associated with the formation, stabilization and enhancement of long-term memories (Walker and Stickgold, 2004)⁠ such that a memory becomes more resistant to change or interference from competing, new memories (McGaugh, 2000). MC is normally observed as an improvement in performance on a given task following sleep that surpasses the asymptotic limit of performance with repeated practice prior to sleep. Sleep-related improvements in various tasks have long been observed, as noted by the Roman scholar, Quintilian, almost 2,000 years ago:

“It is a curious fact, of which the reason is not obvious, that the interval of a single night will greatly increase the strength of the memory, whether this be due to the fact that it has rested from the labour, the fatigue of which constituted the obstacle to success, or whether it be that the power of recollection, which is the most important element of memory, undergoes a process of ripening and maturing during the time which intervenes. Whatever the cause, things which could not be recalled on the spot are easily co-ordinated the next day, and time itself, which is generally accounted one of the causes of forgetfulness, as to strengthen the memory.” (Quintilian, translated by Butler, 1925)⁠

**FIGURE 1:**
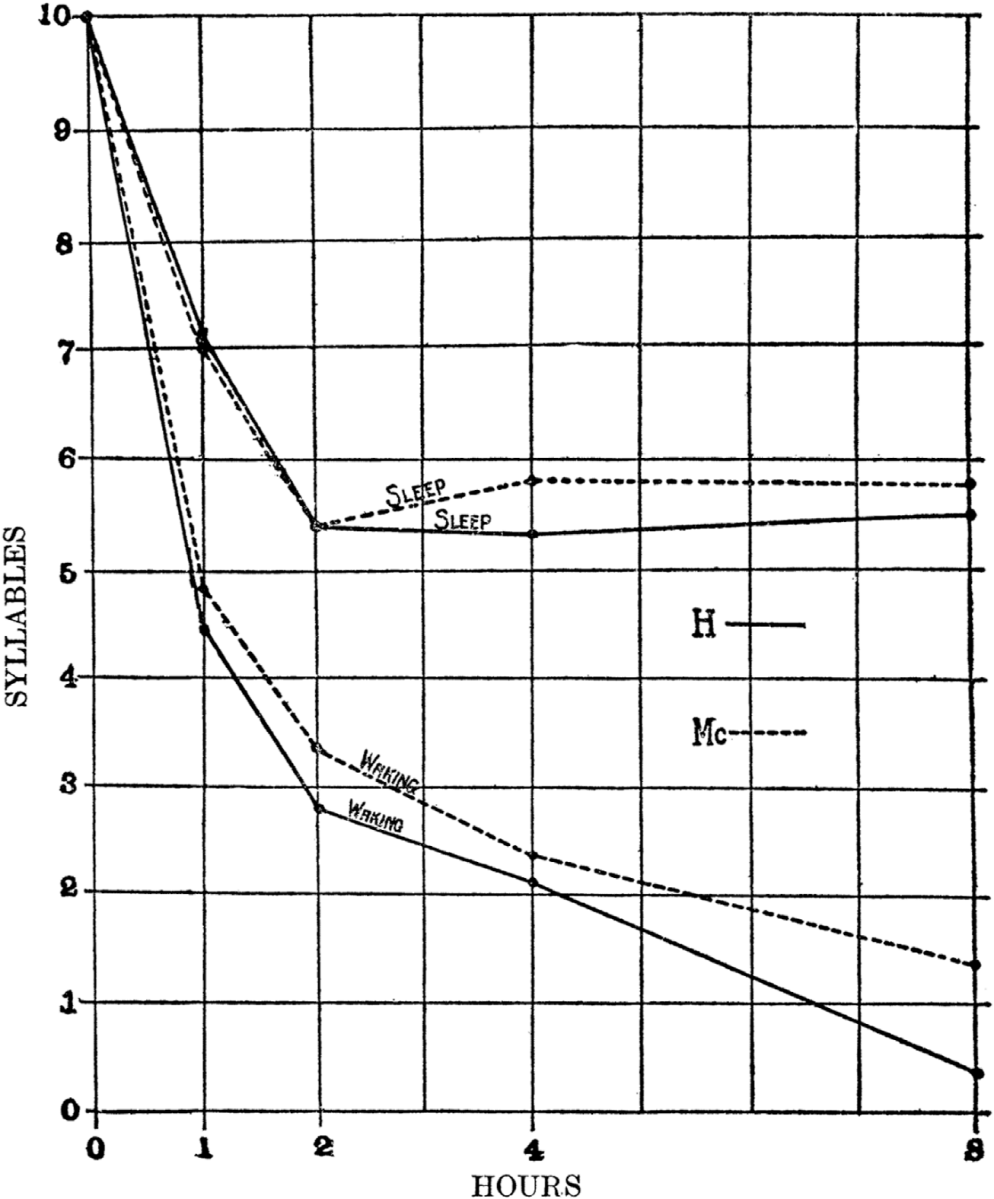
Sleep protects memories against forgetfulness. Two students were given a series of nonsense syllable-pairs and asked to reproduce the series at various latencies spent either awake or asleep. While “Waking”, a continuous trend of forgetting occurred over several hours that was not observed when the time was spent in “Sleep”. In addition, something occurred after 2 h of sleep that blocked forgetting, but the authors did not speculate on the underlying mechanism (from Jenkins and Dellenbach, 1924).

The term “consolidation” was originally proposed more than a century ago to describe the strengthening of memories over time, but without regard to sleep (Müller and Pilzecher, 1900)⁠. The first quantitative evidence that sleep slowed or prevented forgetting was shown in a verbal memory task where subjects memorized lists of nonsense syllable-pairs and then were tested for recall at different latencies spent either awake or asleep (Figure 1) (Jenkins and Dallenbach, 1924)⁠. Consolidation of memories has been observed in numerous neural systems (e.g., visual and auditory tasks, as well as declarative and procedural memory) (Walker and Stickgold, 2004)⁠ and in a wide range of species (Agranoff et al., 1965; Peigneux et al., 2003; Vorster and Born, 2015)⁠. Physiological MC is a complex process involving not just the incorporation of new memories into existing memories, but also the modification of previously established memories (Lechner et al., 1999)⁠⁠.

From a theoretical standpoint, the realization that information could be stored in the weights connecting nodes in artificial neural networks (Rashevsky, 1938)⁠, and the description of an algorithm for how co-active neurons could strengthen those weights laid the groundwork of learning algorithms (Hebb, 1949)⁠, but did not relate learning to sleep or consolidation. Hebb’s work, in particular, is relevant to neural plasticity in regards to epilepsy, because it proposed that persistent changes in neural circuits arose from neuronal co-activation and synchrony, which is a hallmark of seizures. Later work in theoretical neuroscience proposed a mechanism for how neocortex could incorporate new, long-term memories and group similar items into categories (Marr, 1970)⁠ after receiving input from short-term memories that were rapidly formed in hippocampus during behavioral experiences (Marr, 1971). Marr’s work was later extended to show that memories could be encoded as “codons” (or “neural assemblies”, groups of neurons whose activity was mutually reinforcing within a given context) and how iterative consolidation allowed new memories to be formed without over-writing existing memories, allowing modification only of those memory engrams that were presented to the network during the learning process (McNaughton and Morris, 1987; McClelland et al., 1995)⁠. While these and other seminal works laid the theoretical foundations for how neural systems acquire, store, and retrieve new information, few of them mention sleep and very little, if anything, is said about epilepsy or how neural plasticity may respond to seizures. They do, however, provide constraints on the type of neural activity that can be stored through physiological processes; namely, neural assemblies.

Anatomically, the involvement of the hippocampus in learning and memory was demonstrated by the case of “HM”, a young man who underwent epilepsy surgery to treat intractable seizures in 1953 at the of age 27. Following a bilateral, mesial temporal lobe resection, the frequency of his seizures was reduced, his personality was unchanged, and he seemed normal is virtually in all respects, except for one, specific and profound deficit: he could no longer form new memories (“anterograde amnesia”) (Scoville and Milner, 1957)⁠⁠. He could remember details about events that occurred in the days prior to his surgery (for decades, afterwards), but could not form new, declarative memories about daily events or interactions with the people that he met. The surgery removed or damaged multiple brain structures, including anterior portions of hippocampus, amygdala, entorhinal cortex, and part of the left fronto-orbital cortex (Annese et al., 2014)⁠. HM’s learning deficits have been studied and debated extensively for decades, but also have been central in developing our understanding of the anatomical underpinnings of memory and amnesia, and have shown that MC involves a hippocampal-neocortical dialogue (Eichenbaum, 2013)⁠. Combining multiple lines of evidence, the “Two Stage” theory of memory formation proposed that neocortical activity during wakefulness rapidly formed “engrams” in hippocampal circuits that were subsequently reactivated during post-behavioral sleep and projected back into neocortical circuits to create or enhance long-term memories (Buzsáki, 1989; Hasselmo, 1999)⁠.

## Types and mechanisms of consolidation

Because MC occurs during sleep, it is helpful to clarify some definitions regarding sleep that will be relevant for quantifying and understanding consolidation. Human sleep can be grouped into two states: Rapid Eye Movement (REM) and non-REM (NREM), where NREM consists of three stages: N1 and N2 that contain sleep spindles and K-complexes and Slow-Wave Sleep (SWS or N3), which derives its name from the emergence of large-amplitude waves between 0.5–4.0 Hz (Berry et al., 2015)⁠. NREM and REM sleep alternate through the night, normally on a 90 min cycle with REM periods dominating early in sleep and NREM dominating later. Initial observations linking MC and sleep stages came from observations of narcotic overdoses that reduced REM sleep following the overdose leading to “REM rebound”, which was then linked to increased protein synthesis during REM sleep (Oswald, 1969)⁠⁠⁠. Further evidence came from the observation that memory-intensive tasks altered sleep architecture by increasing the proportion of time spent in Slow-Wave Sleep (Gais and Born, 2004)⁠ and that interrupting specific sleep stages disrupted the formation of new memories (Smith and MacNeill, 1994)⁠. While consolidation is observed in both SWS and REM sleep, the specific mechanisms of neural plasticity and the types of memories consolidated may differ in the two conditions (Siegel, 2001; Rasch and Born, 2013)⁠.

**FIGURE 2:**
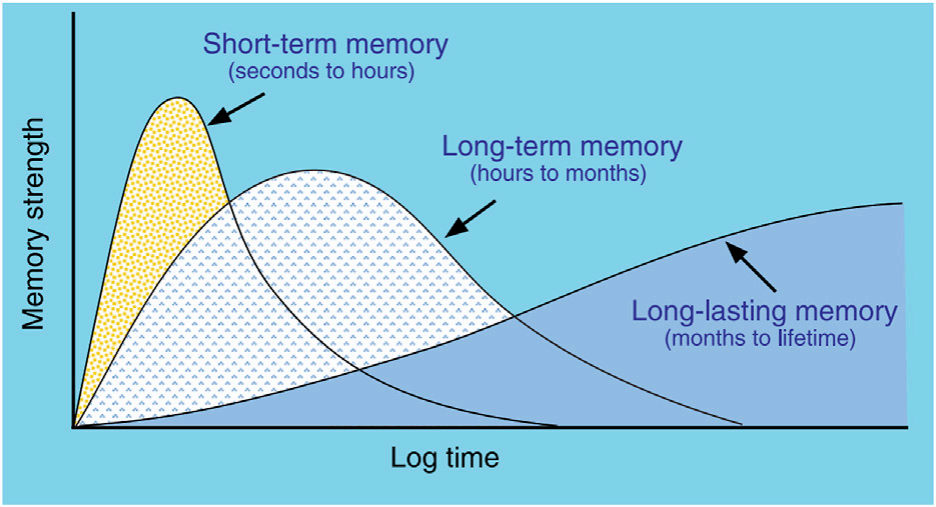
Types of memory linked to different consolidation mechanisms. Short-term memory does not require protein synthesis and appears related to LTP mechanisms. Long-term memory does require protein synthesis and utilizes cellular and systems consolidation. The mechanisms of Long-lasting memory are less clear, but likely involve re-consolidation involving multiple brain structures to enhance memories. (from McGaugh, 2000).

The physiological mechanisms underlying Memory Consolidation (MC) have been the focus of intense investigation for decades and have been shown to include several, distinct mechanisms of consolidation acting on different timescales, each utilizing unique physiological processes (Figure 2) (McGaugh, 2000; Rasch and Born, 2007; Dudai and Morris, 2013)⁠. Much of what is known that distinguishes the various mechanisms of consolidation derives from the methods used to selectively disrupt each of them (Reyes-Resina et al., 2021)⁠. To clarify the nomenclature, we will describe behavioral Memory Consolidation by the two-letter acronym of “MC”, while the different mechanisms thought to underlie MC will use a three-letter acronym that describes the type of “Consolidation of Memory” (xCM). For example, Cellular Consolidation of Memory (CCM) describes changes that occur within hours of an experience, involve the hippocampus and amygdala, involve glutamate AMPA receptors (because they can be disrupted by application of AP5 and CNQX), and are consistent with stimulation parameters that are required for Long-Term Potentiation (LTP) to alter synaptic strength (Bliss and Lømo, 1973; Jerusalinsky et al., 1992; Malenka and Nicoll, 1999)⁠. Systems Consolidation of Memory (SCM) describes protein-synthesis-dependent changes involving multiple brain structures including hippocampus, thalamus, and neocortex that operate on the timescale of hours to days (Squire and Alvarez, 1995; McClelland and Goddard, 1996; Dudai and Morris, 2013)⁠⁠⁠. SCM occurs primarily during sleep and allows the formation of new memories or the strengthening of specific, existing memories without over-writing or interfering with other, existing memories (McClelland et al., 1995)⁠. Re-Consolidation of Memory (RCM) describes how existing memories become “labile” when activity similar to previous activations overlaps existing memories, allowing for modification of those existing memories (Spear, 1973; Lechner et al., 1999; Nader et al., 2000)⁠. Recently, interference with RCM has become an area of therapeutic interest in regards to treating PTSD by helping patients recall traumatic memories in a safe context, thus reactivating those memories in an attempt to modify those memories through consolidation mechanisms in order to lessen the severity of the condition (Taubenfeld et al., 2009; Brunet et al., 2018; Bolsoni and Zuardi, 2019).⁠ Broadly then, CCM refers to the encoding of new memories, SCM refers to the formation of long-term memories utilizing mechanisms requiring protein synthesis, and RCM refers to the modification of existing long-term memories. While all three types may play a role in the consolidation of seizure-related activity, the focus of *in vivo*, electrophysiological studies, to date, has been on SCM and changes observed during sleep.

Systems electrophysiology, both *in vivo* recordings of multiple, single neurons via microelectrodes (radius < 100 μ) and EEG via macroelectrodes, provides a window on the mechanisms of SCM. An early, electrophysiological perspective on the mechanisms underlying SCM came from the observation that CA1, hippocampal neurons that were more active during a behavioral task became more active during subsequent sleep, compared to neurons that were inactive during the task (Pavlides and Winson, 1989)⁠. Ensemble recordings of neurons (i.e., the simultaneous recording of dozens of individual neurons) in rats running on mazes revealed that populations of neurons encoded position in a subset of neurons that were active in a given environment (an “assembly”) (Wilson and McNaughton, 1993).⁠ One possibility is that neural assemblies form a unique “memory index” that becomes associated with a given pattern of neocortical activation through CCM, which might involve LTP (Bliss and Lømo, 1973; Teyler and DiScenna, 1986)⁠. During post-behavioral sleep, the firing of pairs of hippocampal neurons that were co-active during maze-running was found to become more correlated during post-seizure sleep even when the correlation between the pair that existed prior to the behavior was taken into account (Wilson and McNaughton, 1994)⁠. The statistical method used to show this is called partial correlation and will be described more fully in the next section, where it will be applied to neuronal firing around the time of seizure onset and during sleep before and after the seizure. The observation that pairs of neurons that “fire together” during behavior subsequently “wire together” during post-behavioral sleep provides a mechanism for the type of learning described by both Rashevsky and Hebb decades prior, and it also suggests that an integral part of this learning mechanism requires the re-instantiation or “reactivation” of prior activity patterns across populations of neurons. Hippocampal reactivation during sleep drives information flow both across local circuits and distant networks, involving the synchronization of different field potential oscillations in neocortex (“slow oscillations”, <1 Hz), thalamus (spindles, 12–15 Hz), and hippocampus (ripples, ∼80 Hz) where the higher frequency signals “nest” inside of the lower frequency signals (Klinzing et al., 2019; Skelin et al., 2019)⁠. In summary, the theory regarding the mechanism underlying MC is that neural assemblies active in neocortex during experience are projected to hippocampus where CCM forms memory index engrams. During SCM during post-behavior sleep, these engrams are “reactivated” from hippocampus and broadcast to thalamus and neocortical structures, producing permanent alterations in synaptic connectivity in those neocortical structures, establishing new memories, enhancing existing ones, or making existing memories less fragile (Buzsáki, 1989; McClelland et al., 1995; McGaugh, 2000)⁠. The decision-making process that determines which experiences are reactivated (and thus stored to long term memory) while other experiences are forgotten is not understood.

## Seizure-Related Consolidation

Before discussing how MC and the mechanisms of SCM might apply to epilepsy, it is worth taking some time to examine the statistical methods used to quantify persistent, functional changes in populations of neurons recorded *in vivo*: specifically, the statistical technique of partial correlation, which measures correlation between two variables after the effect of a third variable has been discounted. A humorous, teaching example regarding partial correlation asked whether it was statistically justifiable to propose that storks deliver babies by considering three variables: the number of storks, the human birth rate, and human population density (Matthews, 2000)⁠. The number of storks in an area was found indeed to be correlated with the number of births (so perhaps storks do deliver babies!), but that could reflect the possibility that people and storks normally live in close proximity to one another. When the population density of people was discounted using partial correlation, the number of storks and births were shown to be uncorrelated. In the case of SCM, the three variables of interest are the correlation of two events (e.g., the firing of two neurons) at three timepoints: *during* a behavior (e.g., maze running) and during SWS *before* and *after* the behavior. Partial correlation then quantifies the correlation between the events *during* and *after* the behavior given their correlation *before* the behavior. To study persistent changes in functional neural connectivity related to epilepsy, the role of *during* a behavior in analysis of long-term memory formation has been replaced by the minutes *prior to* seizure initiation. If neurons that “fire together” in the minutes *prior to* a seizure and then “wire together” *after* the seizure, then their partial correlation would be expected to increase relative to their correlation *before* the seizure.

Epilepsy and neural plasticity mechanisms have long been thought to be related. Goddard and Douglas (1975)⁠ noted that network changes resulting from kindling were “trans-synaptic” and “widespread”, similar to what was observed in “normal learning”. Normal brain connectivity and oscillations during sleep were proposed to provide a “template” for epilepsy to “hijack” neural plasticity mechanisms, modifying neural circuits to make them more likely to initiate seizures (Beenhakker and Huguenard, 2009)⁠. Halász and Szűcs (2020)⁠ looked at four, common types of epilepsy and proposed specific anatomical, neural systems that could serve as the templates for epilepsy by showing how each system was modified during sleep. This body of work suggested that neural plasticity mechanisms played a role in epileptogenesis, but the actual mechanisms involved were not clear.

**FIGURE 3:**
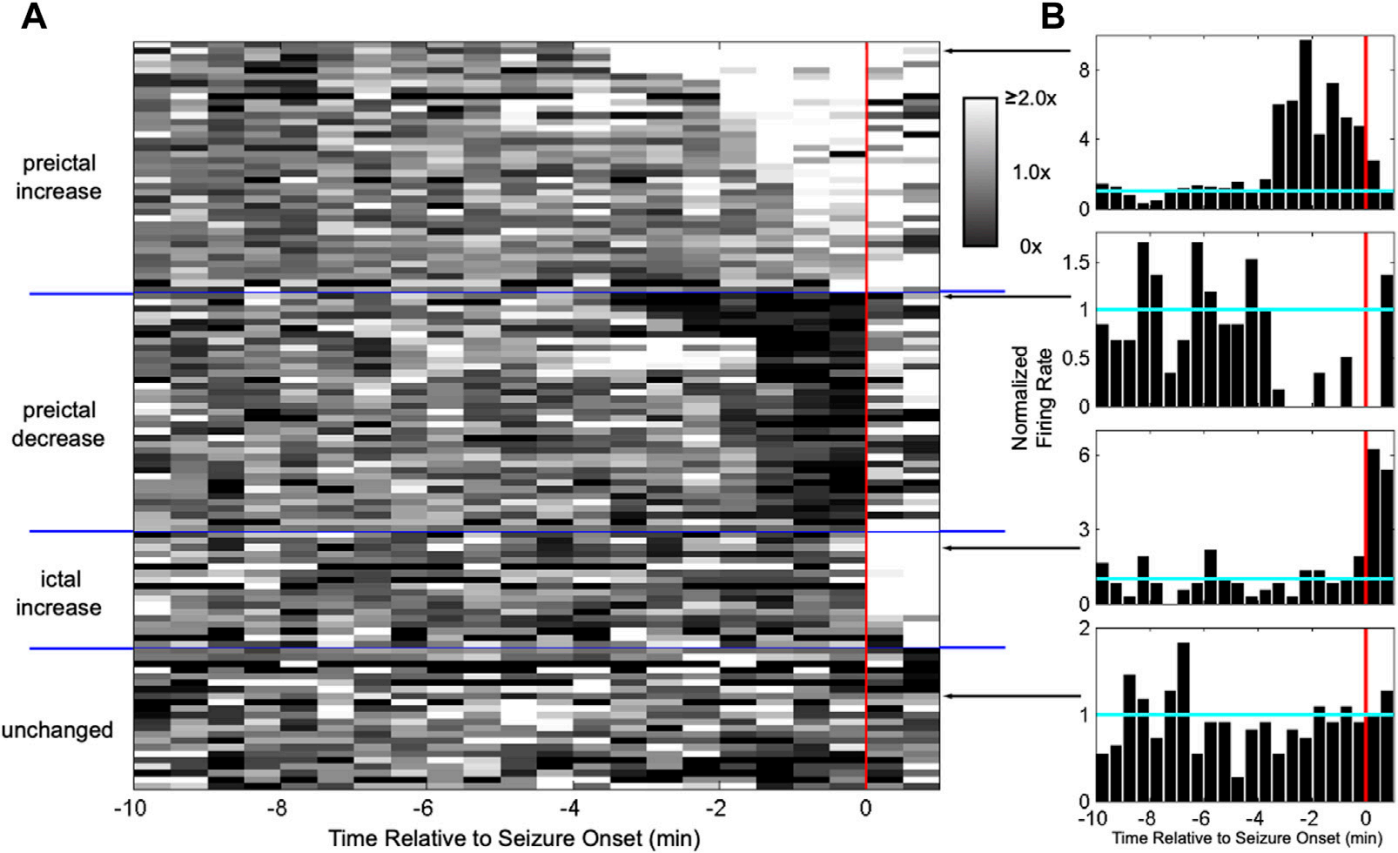
Heterogeneous activation of granule cells at seizure onset resembles neural assemblies. **(A)** Each row shows the normalized firing rate in grayscale for a granule cell in a pilocarpine-treated rat around seizure onset (red line). The firing rate of some granule cells increased starting minutes before onset, while others decreased, others increased only after onset, and others did not change at all. Having a self-reinforcing subset of neurons active during a behavior is known as an “assembly”. **(B)** Examples of responses from individual granule cells. (from Bower and Buckmaster, 2008).

One possibility is that seizures consist of “pathological” signals among neuronal populations; i.e., the neural activity associated with the initiation and progression of seizures is somehow dramatically different than that associated with normal, everyday behavior. This seems reasonable, because the behavioral and cognitive manifestations of seizures can differ so greatly from non-seizure behavior. When the first neuronal ensemble recordings prior to and during the initiation of seizures were obtained, however, the activity patterns across the population of recorded hippocampal dentate gyrus granule cells in pilocarpine treated rats did not differ dramatically from what was observed during normal behavior (Bower and Buckmaster, 2008)⁠. While the firing rate of many granule cells increased, that of others decreased, while that others remained unchanged, similar to what is observed during normal behavior (Figure 3). Neural activity at the initiation of seizures possessed some similarities to those observed during physiological behaviors; i.e., the population of neurons appeared to be a neural assembly. Whether neural assemblies persist throughout the duration of the seizure is difficult to determine using electrophysiology due to the increasing background noise induced by the seizure. Optical imaging of neurons, however, offers a promising approach to this question and it has already shown both differential recruitment of classes of neurons as well as evidence that not all neurons are recruited across the duration of a seizure (Somarowthu et al., 2021; Xiong et al., 2023)⁠.

**FIGURE 4:**
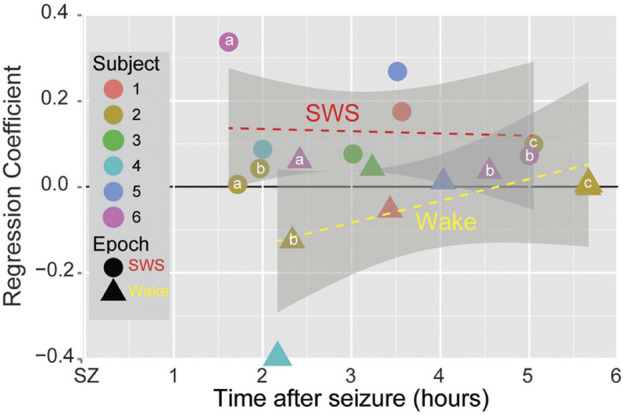
Seizure-Related Consolidation in patients. Circles and triangles show the averaged regression coefficient for all partial correlation coefficients for SWS and Wake (resp.) across all pairs of single neurons for each spontaneous seizure in six subjects as a function of time from the termination of the seizure. Small letters indicate multiple seizures within the same patient (“a” denotes the first seizure, “b” the second, “c” the third). For each seizure, the regression coefficient for SWS was larger than for Wake. Grey regions show the 95% confidence interval. (from Bower et al., 2015).

The observation of neural assemblies at the onset of seizures raised the possibility that these assemblies could be reactivated during post-seizure sleep and thus that neural plasticity mechanisms might treat pathological, seizure-related activity in the same manner as neural assemblies produced by normal behavior. Using the methodology of partial correlation described previously, the *behavior* time epoch was replaced with the *prior to* seizure epoch, correlation coefficients were computed between pairs of neurons during the *prior to* as well as for SWS and Wake epochs immediately preceding and following the seizure, and then partial correlation coefficients were computed for *prior to* ∼ *after* given *before* sleep epochs. Ensemble recordings from patients undergoing intracranial monitoring for epilepsy showed that the neural assemblies present at seizure onset were reactivated during post-seizure SWS, but not during post-seizure Wake (Figure 4) (Bower et al., 2015)⁠.

**FIGURE 5:**
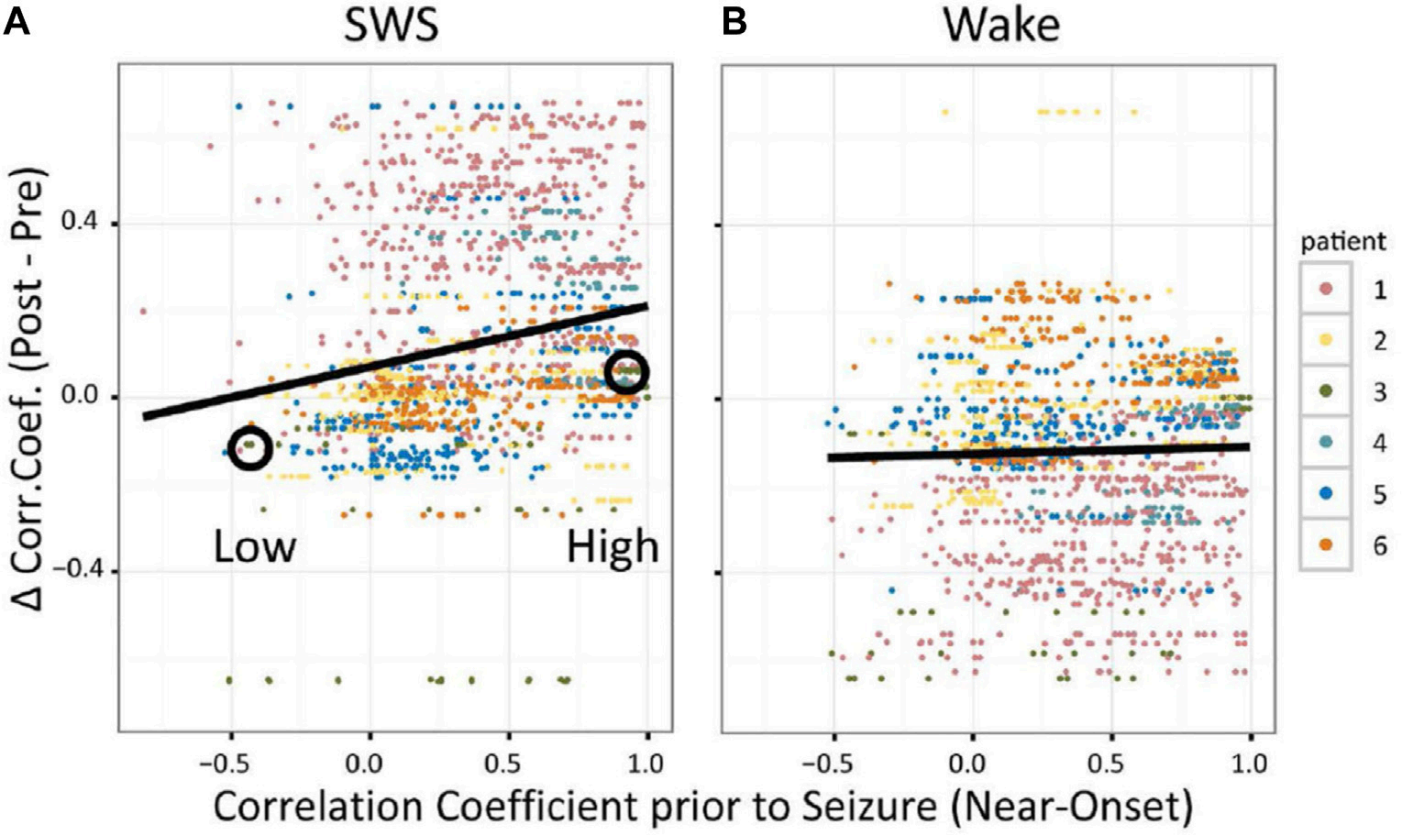
Seizure-Related Consolidation in patients observed in EEG (inter-ictal spikes, IIS). For SWS **(A)** and Wake **(B)**, each dot shows the difference in correlation coefficient (CC) for IIS detected on a pair of macroelectrodes from after versus before the seizure as a function of CC prior to seizure onset. (from Bower et al., 2017).

Normally, the effects of neural plasticity are subtle and difficult to observe (Guskjolen and Cembrowski, 2023)⁠, even at the level of ensemble recordings of populations of single neurons. The clarity of SRC in pairs of neurons following seizures, however, suggested that persistent changes in circuits might not only be observable as changes in synchrony at the level of neurons, but might also be observable as persistent changes in field potentials. When correlation coefficients for IIS detected on different macro-electrodes were computed and then used as inputs into a partial correlation analysis (as with multiple, single neurons), reactivation was observed for IIS in post-seizure SWS, but not post-seizure Wake (Figure 5) (Bower et al., 2017)⁠. Because EEG field potentials are thought to reflect the spatially summed, distal inputs to dendritic spines (Herreras, 2016)⁠, reactivation of seizure-related activity might not just reflect local changes in neural circuits, as described previously by theories emphasizing changes in local circuitry, but also by increased changes from distal, input circuits, thus linking the original description of NTE to neural plasticity mechanisms associated with epileptogenesis.

## Discussion

**FIGURE 6:**
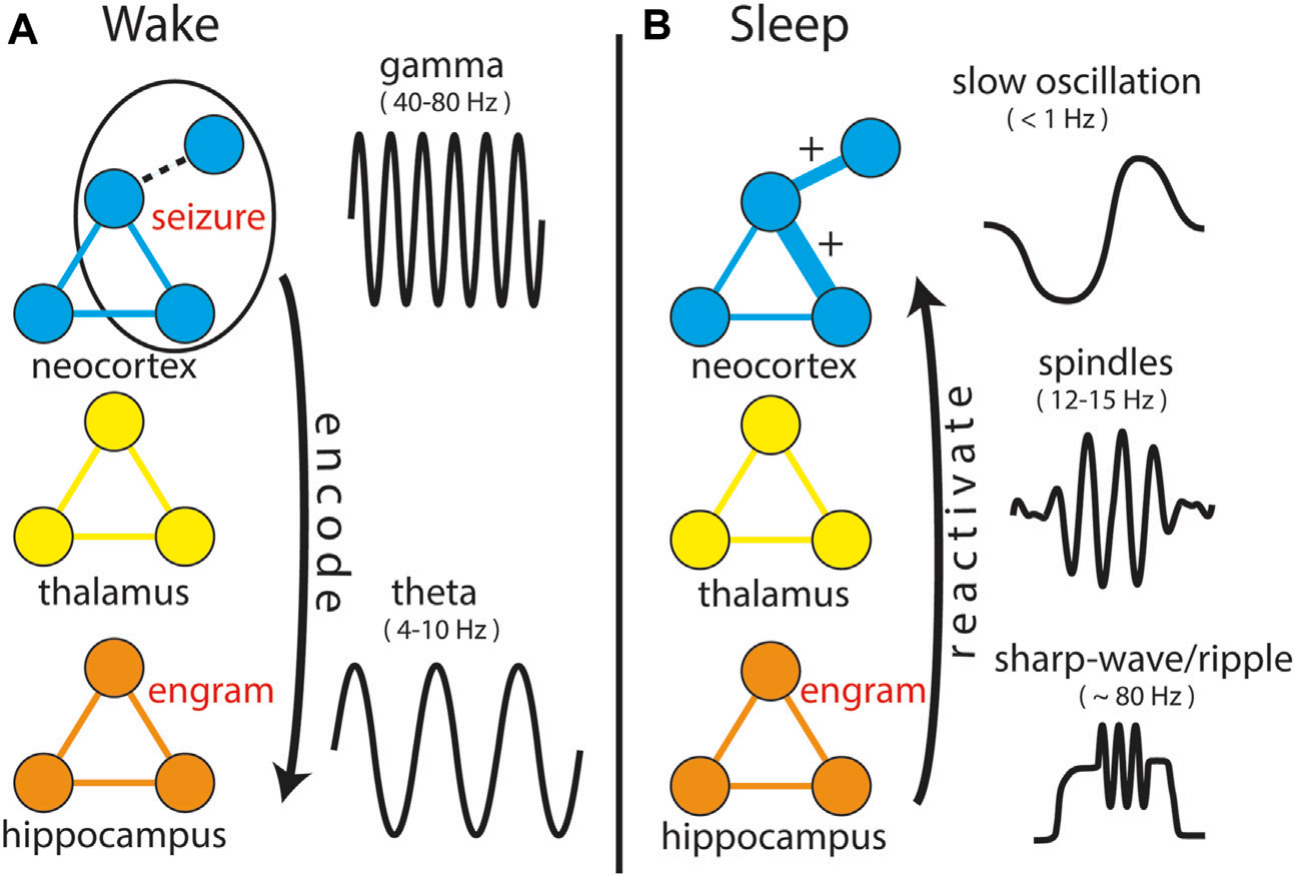
Expanding the Network Theory of Epilepsy. In both panels, prominent EEG signals (not shown to scale, but displayed for clarity of differences) specific to both anatomical structure and behavioral state are shown. **(A)** During “Wake”, a “seizure” involves three neurons, including one that is weakly connected to the network (dotted line). This activity is projected to hippocampal circuitry (arrow) in the same manner as normal behaviors, encoding an “engram” of the seizure. During normal behaviors, encoding as aided by synchronization of neocortical “gamma” and hippocampal “theta” oscillations, though it remains unclear how encoding occurs for seizure-related activity. **(B)** During “sleep”, the engram is reactivated and projected back into the structures and circuits involved in the seizure, strengthening connections between neurons that were co-active, including the weakly connected one, which now becomes a strong connection (“+”).

Pulling together multiple perspectives, we have reviewed how mechanisms underlying MC provide a relatively unexplored link between sleep and epilepsy, that CCM and SCM appear to underlie this linkage, that neural assemblies and synchrony of field potentials (IIS) that arise at the initiation of seizures are reactivated during post-seizure sleep, and that this reactivation (similar to physiological learning) is associated with persistent changes in neural activity, which has been labeled “Seizure-Related Consolidation” (SRC) (Bower et al., 2015; 2017)⁠. The observation that reactivation and subsequent, persistent, functional changes were observed both at the level of single neurons (i.e., local circuits) and IIS (i.e., distal inputs) suggests structures both local and distal to recordings showed involvement in post-seizure, neural plasticity. The observed results would require the reactivation of seizure-related activity in multiple brain structures, even those that may not have been involved in the seizure itself (Figure 6) and would also require that those structures function normally to allow SCM to occur. Evidence in support of this is found in patients with focal cortical dysplasia (FCD), a congenital abnormality involving improper neural migration during development in a specific brain region. Age of onset for seizures was found to be related to the affected neural system (Macdonald-Laurs et al., 2024)⁠ where the latest age of seizure onset occurs in patients whose lesions are located in limbic structures (Cohen et al., 2022), suggesting impaired SCM delayed the onset of seizures⁠. Children with limbic FCD also suffer from impaired memory (Rzezak et al., 2014)⁠, suggesting the FCD in limbic structures impairs memory formation processes, such as memory consolidation, supporting the hypothesis that MC is involved in epileptogenesis. This would also provide an explanation for why the “quiet period” between an initial, seizure-inducing insult (e.g., trauma, fever) and subsequent development of epilepsy does not lead to a sudden onset of seizures, but rather consists of a “continuous process” during which atypical neural activity is observed (Dudek and Staley, 2011). Sub-clinical reactivation of epileptiform activity arising from the initial insult that is insufficient to initiate seizures at first could iteratively build over months to years, occurring on a similar time scale to “long-lasting memory” previously shown in Figure 2, until reaching a threshold where seizures begin to occur.

Several confounds could affect studies of consolidation and epilepsy. For partial correlation analyses, the epoch when neural activity involved in producing a non-stressful behavior (e.g., learning a motor or verbal task) was replaced by an epoch containing pre-ictal activity occurring within minutes of an impending seizure, which can be a stressful and anxiety-producing experience and is associated with heightened levels of cortisol and other compounds (Cano-López and González-Bono, 2019)⁠ that are known to affect learning (Vogel and Schwabe, 2016)⁠. A second confound is sleep deprivation that often accompanies seizures. Sleep deprivation is known to affect consolidation mechanisms, as well as modifying sleep architecture; e.g., altering the proportion of time spent in various sleep stages (Elmenhorst et al., 2008)⁠. In addition, sleep deprivation (which can follow seizures) can alter the duration of sleep by inducing “rebound” sleep, which lasts longer and spends more time in REM (Berger and Oswald, 1962; Lucidi et al., 1997; Rechtschaffen et al., 1999)⁠. An additional confound regarding statistical analysis when using partial correlation to study epileptogenesis is that seizures present a “moving target” in that the goal of the learning continually changes; if each seizure activates a unique population of neurons, then the cumulative effects of reactivation will not necessarily be additive. Unlike the learning of a behavioral task where improvement asymptotically approaches a fixed goal, change across a series of seizures has no goal, which adds to the difficulty of attempting to “unlearn” a seizure (Hsu et al., 2008)⁠. This change can only be measured as a series of related events where we can only determine whether more changes in correlation (pairs of neurons or IIS that are positively correlated prior to the seizure show increased correlation in post-seizure sleep) occur than would be expected by chance.

Considering SRC as a mechanism of epileptogenesis that extends the Network Theory of Epilepsy is parsimonious with the mechanisms of learning and memory; i.e., SRC as an epileptogenic mechanism does not require any new mechanisms or properties that are uniquely required for epileptogenesis and that are not observed in any other type of activity. Rather, SRC suggests that epilepsy is a natural extension of the interconnected networks of the brain and the processes by which those networks are modified. Therapies that disrupt SRC by disrupting SWS and/or REM following seizures might disrupt epileptogenic changes, reducing future frequency and severity of seizures similar to the reduction in symptoms for PTSD patients. While disrupted sleep would certainly cause patients to be tired the next day, it should be noted that sleep the following night could proceed normally without impacting the therapeutic benefits disrupting SRC; consolidation is thought to primarily occur on the night following the behavior or seizure. Perhaps the root mechanisms of epilepsy have been uniquely evasive because they are not linked to a specific pathology, but rather result from a progression of typical, physiological changes that escape typical, physiological bounds.

NTE diverged from a long history of assumptions regarding the initiation of seizures that centered on a “focus” of “sick” neurons, circuits, or oscillations that cause seizures to differ so dramatically from typical behavior. Perhaps difficulties in understanding how epilepsy progresses have persisted because epilepsy does not necessarily arise or worsen due to unique pathological mechanisms present only after seizures appear, but rather that epilepsy arises from normal, physiological processes placing networks of interconnected brain structures into vulnerable, neurological states that are not easily measured or categorized. Since NTE was proposed over 20 years ago, several avenues of research have shown that the progression of epilepsy involves more than just those portions of brain tissue that are involved in the generation of seizures, several of which we have covered in this review. The once well-accepted concept of a seizure “focus” from which all seizures are initiated and within which all neurons become hyperactive, itself, has been challenged, both mechanistically (Stead et al., 2010; Bower et al., 2012; Toyoda et al., 2013)⁠ and clinically (Piper et al., 2022)⁠. The Network Theory of Epilepsy has become increasingly important over the past 20 years, not just in terms of expanding our views on how seizures start and spread, but also in other issues related to epilepsy, such as how epilepsy develops during the “quiet period” and continues to progress. It seems reasonable to imagine that NTE will only continue to gain in importance over the next 20 years.

## Data Availability

The original contributions presented in the study are included in the article/Supplementary Material, further inquiries can be directed to the corresponding author.
